# Acceptability, Feasibility, and Uptake of COVID-19 Antigen Rapid Diagnostic Self-Testing at the Community Level in Tanzania

**DOI:** 10.4269/ajtmh.23-0732

**Published:** 2024-11-26

**Authors:** Grace W. Mwangoka, Ali M. Ali, Mwifadhi Mrisho, Abdallah Mkopi, Muhidin Mahende, Hajirani M. Msuya, Silas G. Temu, Paul Kazyoba, Michael G. Mihayo, Omar Juma, Ali Hamad, Said A. Jongo, Omar Lweno, Anneth Tumbo, Sara S. Mswata, Anne Hoppe, Pallavi Dani, Salim Abdulla

**Affiliations:** ^1^ Ifakara Health Institute, Dar es Salaam, Tanzania;; ^2^ National Institute for Medical Research, Dar es Salaam, Tanzania;; ^3^ FIND, Geneva, Switzerland;; ^4^ Elizabeth Glaser Paediatric AIDS Foundation (EGPAF), Geneva, Switzerland

## Abstract

The rapid diagnosis of coronavirus disease 2019 (COVID-19) is critical for comprehensive public health response strategies, and self-testing with antigen rapid diagnostic tests (Ag-RDTs) presents opportunities to test in hard-to-reach communities. Therefore, we evaluated the acceptability, feasibility, and uptake of Ag-RDT self-testing at the community level in Tanzania. From June to October 2022, symptomatic individuals or those with recent contact with a known or suspected COVID-19 patient were offered assisted testing and self-testing within mining communities and at transport hubs. This study included a cross-sectional survey before and after implementation. Participants were assessed for their acceptability and uptake of the nasal Ag-RDT self-test and their preference for nasal Ag-RDT self-testing. The survey data were collected in Open Data Kit, whereas the Ag-RDT results in the community were recorded by using the COVISUSPECT Mobile Application. Data analysis was performed by using STATA and R Statistical Software. A total of 538 individuals were screened, and 454 (84.4%) consented to be tested. The preference for self-testing was relatively low (33%), and the majority of participants (67%) opted to be assisted by a healthcare professional. Of the participants who opted for testing, 149 (32.8%) were able to self-test. Generally, there was no major difference in the various assessed parameters between the baseline and end-line surveys. The results from fitting multiple logistic regression indicated that after controlling for age, participants living in Dodoma were significantly less likely to opt for self-testing (odds ratio = 0.54; *P*-value = 0.023) compared with those living in Dar es Salaam. There was no significant difference in self-testing between participants living in Mara and those living in Dar es Salaam (odds ratio = 0.7; *P*-value = 0.179). After controlling for region, older (≥40 years) participants were significantly less likely to self-test compared with participants aged 18 to <40 years (odds ratio = 0.47; *P*-value = 0.002). The intervention was well-accepted in all areas in which Ag-RDTs were deployed. Our findings can therefore support the Ministry of Health by increasing accessibility to severe acute respiratory syndrome coronavirus 2 testing in the hard-to-reach communities in response to the next COVID-19 wave.

## INTRODUCTION

There is still no consensus on the best approach for managing severe acute respiratory syndrome coronavirus 2 (SARS-CoV-2) outbreaks, especially in low- and middle-income countries (LMICs). The feasibility of eliminating SARS-CoV-2 is low,^1^ and the virus continues to have high transmission potential.^2^ Furthermore, waning protection from previous infections and vaccination, combined with new, highly infectious circulating variants might make it difficult to eliminate the virus through herd immunity.^3^ It is therefore essential to mitigate the consequences of future SARS-CoV-2 outbreaks by developing strategies that are expected to mitigate transmissions in hard-to-reach communities.^4^^,^^5^

During the pandemic, Tanzania was committed to the rollout of coronavirus disease 2019 (COVID-19) antigen rapid diagnostic tests (Ag-RDTs) in all public health facilities and communities to expand the scope and coverage of COVID-19 diagnosis in resource-constrained settings.^6^ It has been shown in high-income countries that COVID-19 self-testing is feasible and delivers reliable results.^7^^,^^8^ Specifically, there was no difference in the quality of results between a test performed by a professional nurse or parent of a child at home.^8^ Self-testing can therefore expand COVID-19 testing services to benefit vulnerable populations in hard-to-reach communities.^9^

In early 2022, there was limited evidence on the usability and acceptability of Ag-RDT-assisted testing or self-testing from studies conducted in LMICs.^10^^,^^11^ Yet, understanding the local sociocultural settings and adaptation of Ag-RDT testing strategies to the local context was critical in ensuring the introduction and scale-up of Ag-RDT testing. Our project aimed to address these gaps by collecting evidence of the acceptability and feasibility of Ag-RDT self-testing at the community level in Tanzania.

## MATERIALS AND METHODS

### Site selection and study population.

Three regions (Dar es Salaam, Ilala District; Dodoma Town; and Mara, Tarime District) representing country zonal areas participated in this operational research. Within each of these districts, one administrative ward with at least 10,000 people was selected as an intervention site, and another site with a similar population size was selected as a control site. Meetings with the regional district administrative authorities were conducted to select the participating wards. The ward selection was based on the availability of healthcare facilities (HCFs) and two community healthcare workers (HCWs) per village, nearby transport or mining hubs with a larger movement of people, a market nearby, and other transport, like taxis or motorcycles. Specifically, in Mara, a gold mining camp (Barrick) was selected. In Dodoma and Dar es Salaam, terminals for town buses (daladala) were chosen. These were important for passengers commuting from different parts of the cities. Bodaboda (motorcycle), and bajaj (tricycle motorcycle) drivers parking near these bus terminals were also included.

Individuals over 18 years of age living in the study areas who met the Ministry of Health (MoH) case definition for COVID-19 testing and agreed to be tested were eligible to participate in the study. To meet the MoH case definition algorithm, individuals needed to have symptoms indicative of COVID-19 or recent contact with a known or suspected COVID-19 patient for testing. Testing in the health facilities and community was based on the same MoH case definition throughout the study period.

### Study design.

The operational research study involved both intervention and control areas. Within control areas, eligible individuals only received Ag-RDT testing at HCFs. There was no community Ag-RDT testing or self-test test distribution within these areas. No information, communication, or education campaigns on Ag-RDT testing were provided in the control area. During the implementation, convenient sampling was used to enroll participants, in which eligible individuals were offered COVID-19 self-testing (an individual able to collect, process, and interpret the result) and Ag-RDT-assisted testing (an individual tested by an HCW) at testing stations in communities using workplace peer-to-peer educators and other influencers, as well as community HCWs. The same testing protocol based on the instructions for use (IFU) translated into Swahili was used by both groups (assisted and self-testing). Unfortunately, we were unable to evaluate the impact of the intervention on SARS-CoV-2 transmission by comparing transmission rates in the intervention and control areas because infection rates were too low in both areas at the time the study was conducted.

Individuals who tested positive were educated on COVID-19 interventions, such as self-isolation, and were provided with masks and sanitizers for their use. Those with symptoms were referred to the nearby HCFs for further management, as needed. Individuals who tested negative or who did not meet the criteria for testing were either advised to get vaccinated or offered vaccination against SARS-CoV-2 at testing stations.

### Cross-sectional survey.

A structured questionnaire was used to collect quantitative data. A cross-sectional survey was conducted before and at the end of the implementation period to determine the feasibility, acceptability, and uptake of the self-test in the selected communities. The interval between the baseline and end-line surveys was 3 months. The surveys were conducted in June and September 2022, but the implementation of the community testing was from July to October 2022. A total of 1,200 participants were included in the baseline and end-line surveys, of whom 200 were surveyed from the intervention and 200 were surveyed from the control communities of each of the three regions. The total number of households per village, as provided by the village or ward leader, was documented. Households with individuals aged 18 years and older were eligible to participate in the cross-sectional survey. Households from the study communities were randomly selected using the RANDBETWEEN function in Microsoft Excel (Microsoft Corporation, Redmond, WA). These required one adult to meet the MoH case definition. In the event that an individual meeting the MoH case definition refused to participate, the next household was selected from the random list. All enrolled participants provided written consent for participation in the survey. Sociodemographic information, knowledge, and attitudes concerning COVID-19, Ag-RDTs, and self-testing, as well as socioeconomic and vaccination status, were collected for all participants. During the baseline survey, follow-up phone calls were made to all participants to clarify any issues and confirm whether, where, and how testing was performed. The survey was conducted under supervision, and the data manager was responsible for data management, quality, and assurance. The survey data were collected in Open Data Kit (ODK; v2021.2.0, University of Washington). The survey was piloted before a test to clarify the developed system.

#### Testing materials, data collection, and data management.

The nasal SARS-COV2 Ag-RDT Flowflex™ COVID-19 Antigen Home Test from ACON (ACON Labs Inc., San Diego, CA), with a sensitivity of 93% and specificity of 100% (https://www.fda.gov/media/152698/download), was used to perform the testing.

In the community, the demographic information of the patients, results, and vaccination statuses was documented in the COVISUSPECT Mobile Application (Dar es Salaam, Tanzania) using electronic unstructured supplementary service data (USSD), an Android-based data capture application. The sociodemographic data captured included sex, age, occupation, educational level, COVID-19 testing history, COVID-19 symptoms, medical history, and signs and symptoms. Furthermore, questions on acceptability, willingness, and the ability to self-test, as well as knowledge of the use of electronic USSD were asked. The submitted data were reviewed daily to ensure the quality of the collected information. Participants who could self-test and enter their results in USSD were allowed to use their phones to increase their privacy. Additionally, all quantitative data in the survey were captured in the ODK. All data generated from community testing were communicated to the MoH.

### Quantitative data analysis.

We performed a descriptive analysis of the primary outcomes of feasibility, acceptance, and uptake of the Ag-RDT self-test at the community level. We compared the results of the baseline and end-line surveys in both the intervention and control areas. Categorical data were summarized by using frequency counts, and percentages and continuous variables were summarized by using mean and SD. A χ^2^ test was used to test the association between categorical values. To evaluate participants’ knowledge, multiple choices were given for each question, and each response was scored to obtain an average for each particular question. An average score of 50% and above was considered knowledgeable about the asked question. Scoring was also conducted for knowledge of symptoms of the disease, how the disease is spread, and risk groups. Univariate and multivariate logistic regression were used to assess the risk factors influencing the preference for self-testing versus assisted testing. A variable with a *P*-value of less than 20% at univariate logistic regression was considered for multivariate logistic regression. A backward elimination, with the help of a likelihood ratio test at a *P*-value of 5%, was used to keep the variable in the final model. The analysis was performed by using STATA (version 18; Stata Corp, College Station, TX), and R Statistical Software (version 3.4.3; The R Foundation, Vienna, Austria) was used for graphical analysis.

## RESULTS

**Figure 1.**
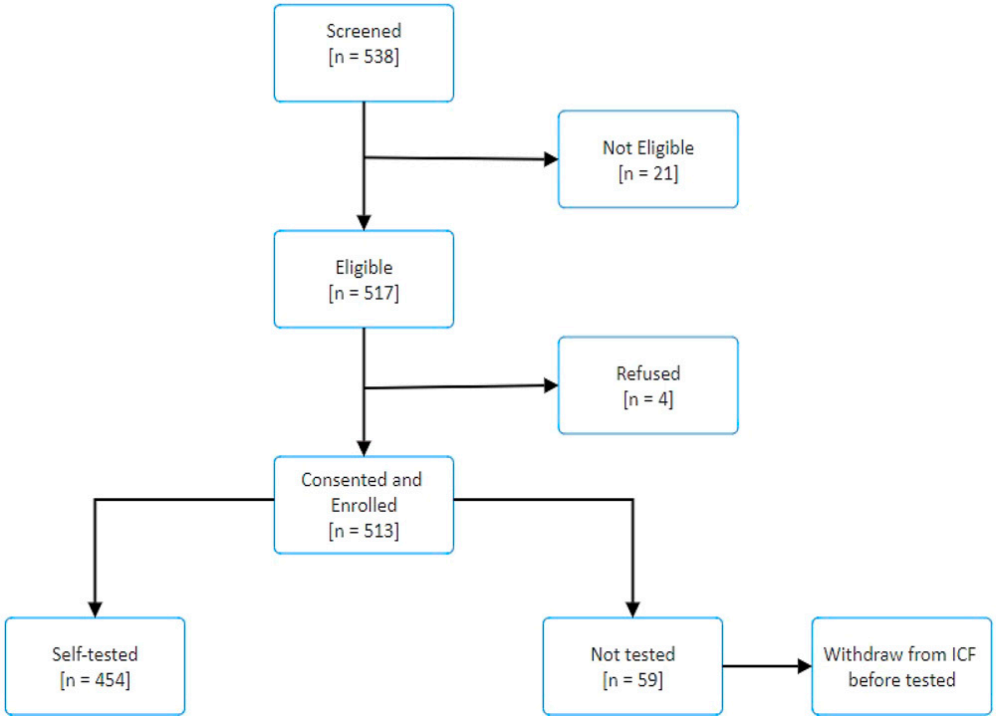
Participant flowchart diagram.

The study was conducted from June 2022 to October 2022 (4 months). A total of 538 individuals were screened by using the MoH COVID-19 testing criteria. Of those screened, 21 (3.9%) were not eligible for testing, and four (0.7%) refused testing. The participant flowchart is presented in Figure 1. The uptake in all regions was good, and only four (0.7%) participants, two from Dar es Salaam and two from Mara, refused testing. Table 1 presents the baseline information of the tested study participants. The majority (266 [58.6%]) of participants tested came from the Dar es Salaam region. In all regions, males and younger individuals (aged 18 to <40 years) tested more frequently. Of those tested, only 170 (37.4%) participants completed primary school education, and 37 (8.2%) of participants attended up to secondary school. Of those who did not complete a primary school education, 206 (45.4%) participants were able to read but not write. Nineteen (4.2%) participants indicated that they were illiterate.

**Table 1:** Characteristics of study participants by site

Variable	Dar es Salaam	Dodoma	Mara-Tarime	Total
Participant tested	*n* = 266	*n* = 93	*n* = 95	454
Sex				
Male	218 (82.0)	69 (74.2)	65 (68.4)	352 (77.5)
Female	26 (9.8)	24 (25.8)	30 (31.6)	80 (17.6)
Missing	22 (8.3)	0 (0)	0 (0)	22 (4.9)
Age (years)				
18 to <40	179 (67.3)	64 (68.8)	55 (57.9)	298 (65.5)
≥40	65 (24.4)	29 (31.2)	40 (42.1)	134 (29.5)
Missing	22 (8.3)	0 (0)	0 (0)	22 (4.9)
Highest educational level attained				
Cannot read and write	1 (0.4)	4 (4.3)	14 (14.7)	19 (4.2)
Can read only	110 (41.4)	46 (49.5)	50 (52.6)	206 (45.4)
Primary	122 (45.9)	22 (23.7)	26 (27.4)	170 (37.4)
Secondary	11 (4.1)	21 (22.6)	5 (5.3)	37 (8.2)
Missing	22 (8.3)	0	0	22 (4.9)
Occupation				
Student	1 (0.4)	0	4 (4.2)	5 (1.1)
Employed	20 (7.5)	17 (18.3)	10 (10.5)	47 (10.4)
Unemployed	16 (6.0)	8 (8.6)	3 (3.2)	27 (6.0)
Self-employed	206 (77.4)	63 (67.7)	67 (70.5)	336 (74.0)
Health medical personnel	1 (0.4)	3 (3.2)	0	4 (0.9)
Others	0	2 (2.2)	11 (11.6)	13 (2.9)
Missing	22 (8.3)	0	0	22 (4.9)

Values are reported as *n *(%). Percentages can be more or less than 100 because of rounding errors. Some participants who might not have attended school can read or can go to school but cannot read and write or can read only. Of those who agreed to participate, 59 refused to be tested and withdrew during the process.

### Acceptability of COVID-19 self-testing.

**Figure 2.**
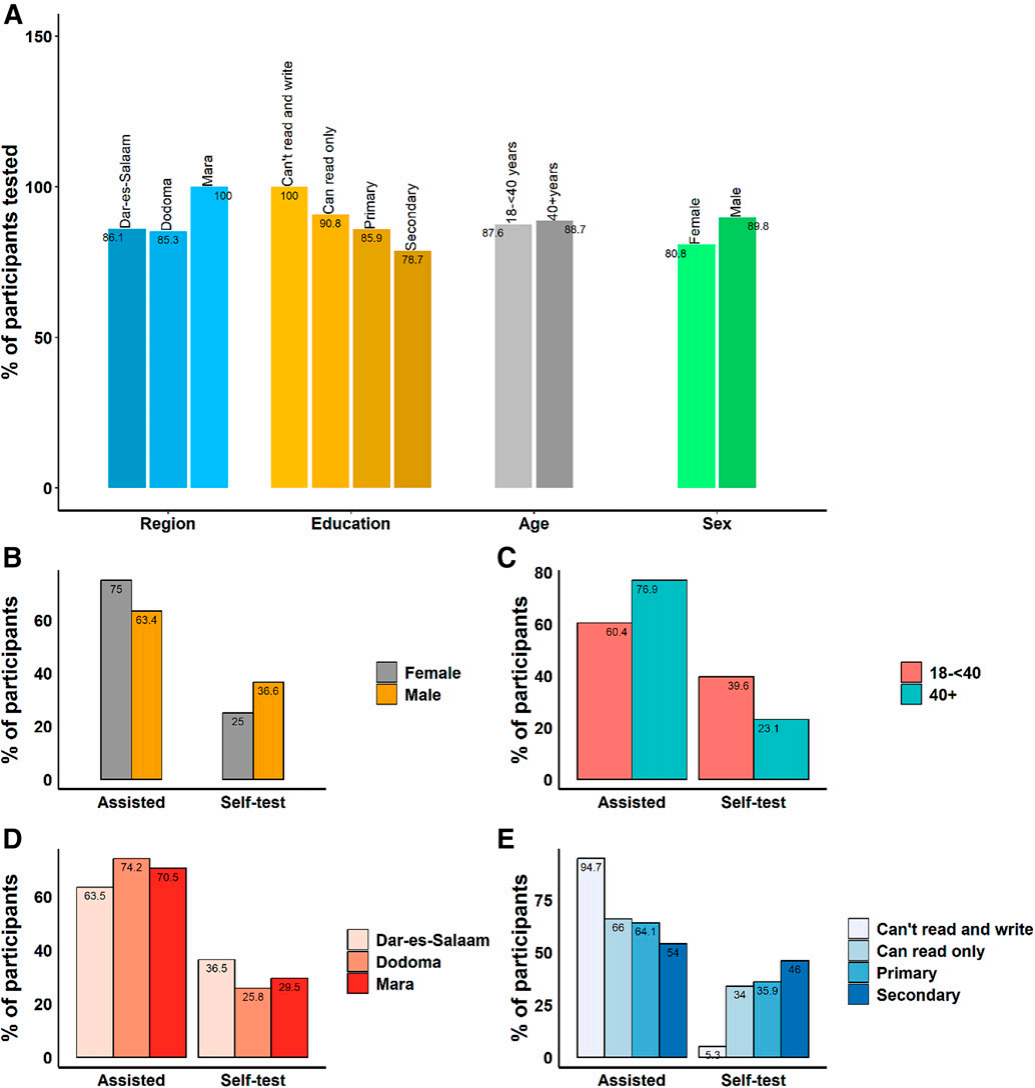
Participants tested at the tent and in the community. (**A**) The proportion of participants tested in each category. The percentages of participants tested was obtained from the total number of participants screened. Percentages were calculated to each category compared those tested and not tested. (**B–E**) The proportions of participants self-tested or assisted by sex (**B**), age (**C**) region they were living (**D**) and level of education (**E**).

Of 513 participants who met the eligibility criteria for testing and agreed to participate, 454 (88.5%) were tested, and 59 (11.5%) were not tested because they refused and withdrew during the process. In the Dar es Salaam and Dodoma regions, 86.1% and 85.3% of eligible individuals tested, respectively, whereas in the Mara region, all eligible individuals tested. Similar testing proportions were seen in the different age categories (Figure 2A).

The confidence to self-test was low, and the majority of those tested were assisted by an HCW. Only 149 (32.8%) of the total participants tested themselves. The number of participants who self-tested was 97 (36.5%), 24 (25.8%), and 28 (29.5%) in Dar es Salaam, Dodoma, and Mara, respectively (χ^2^ *P*-value = 0.125). Twenty (25.0%) females opted to self-test, whereas 129 (36.6%) males self-tested (χ^2^ *P*-value = 0.048). A higher proportion (39.6%) of the younger age category (aged 18 to <40 years) opted to self-test, whereas for those aged ≥40 years and above, only 23.1% self-tested (χ^2^ *P*-value = 0.001). More (46%) participants who had a secondary education tested themselves, compared with 70 (34%) participants who were only able to read. Only one (5.3%) illiterate participant opted for self-testing (Table 2; Figure 2B–E). A multivariate logistic regression presents the results of an adjusted odds ratio, with the region and age being the only variables included. The results from the multiple logistic regression indicated that only the ages of the participants and the regions they came from were associated with self-testing versus assisted testing. After controlling for age, participants living in Dodoma were significantly less likely (46%) to opt for self-testing (odds ratio = 0.54; *P*-value = 0.023) compared with those living in Dar es Salaam. On the other hand, participants living in Mara were 30% less likely to self-test compared with those in Dar es Salaam, but this was not statistically significant (odds ratio = 0.7; *P*-value = 0.179). After controlling for the region, older (≥40 years) participants were significantly less (53%) likely to test themselves compared with participants aged 18 to <40 years (odds ratio = 0.47; *P*-value = 0.002; Table 2).

**Table 2:** Univariate and multivariate logistic regression on the factor affecting coronavirus disease 2019 self-testing

Variable	Self-Test	Assisted	Univariate Logistic Regression			Multivariate Logistic Regression		
			Odds Ratio (SE)	*P*-Value	95% CI	Odds Ratio (SE)	*P*-Value	95% CI
Sex								
Male	129 (36.6)	223 (63.4)	Reference category			–	–	–
Female	20 (25.0)	60 (75.0)	0.58 (0.16)	0.05	0.33–1.00	–	–	–
Missing	0 (0)	22 (100)	–	–	–	–	–	–
Region								
Dar es Salaam	97 (36.5)	169 (63.5)	Reference category			–	–	–
Dodoma	24 (25.8)	69 (74.2)	0.61 (0.16)	0.063	0.36–1.03	0.54 (0.15)	0.023	0.31–0.92
Mara	28 (29.5)	67 (70.5)	0.73 (0.19)	0.22	0.44–1.21	0.70 (0.18)	0.179	0.42–1.18
Age (years)								
18 to <40	118 (39.6)	180 (60.4)	Reference category			–	–	–
≥40	31 (23.1)	103 (76.9)	0.46 (0.11)	0.001	0.29–0.73	0.47 (0.11)	0.002	0.30–0.76
Missing	0 (0)	22 (100)	–	–	–	–	–	–
Highest educational level attained								
Cannot read and write	1 (5.3)	18 (94.7)	Reference category			–	–	–
Can read only	70 (34.0)	136 (66.0)				–	–	–
Primary	61 (35.9)	109 (64.1)	1.21 (0.26)	0.367	0.80–1.85	–	–	–
Secondary	17 (46.0)	20 (54.0)	1.84 (0.66)	0.089	0.91–3.73	–	–	–
Missing	0 (0)	22 (100)	–	–	–	–	–	–

SE = standard error of the mean. Values are reported as *n* (%). *P*-values were derived from χ^2^ or Fisher’s exact tests when more than 20% of cells had expected frequencies of <5. Multivariate logistic regression presents the results of adjusted odds ratios, with the region and age being the only variables included.

A total of 287 (63.2%) community-based Ag-RDT test results were sent to the central database. Among the results sent, 145 (50.5%) were sent by the participants themselves, and 142 (49.5%) were sent by the assistants. A total of 125 (51.9%) males were confident to send the results themselves, whereas only 18 (40.9%) of the females opted to do so (χ^2^ *P*-value = 0.181). In the Mara region, there were significantly (*P*-value <0.001) more participants 49 (64.5%) who sent the results themselves compared with the Dar es Salaam 83 (50.9%) and Dodoma 13 (27.1%) regions. There was no statistical difference (χ^2^ *P*-value = 0.672) in the confidence to send results between the younger age category (aged 18 to <40 years) and those aged ≥40 years (100; 51.0% versus 43; 48.3%). Participants with higher education were more likely (χ^2^ *P*-value = 0.007) to send results themselves compared with participants who did not go to school (Table 3).

**Table 3:** Proportion of participants at tent who opted to send results by themselves

Variable	Self-Send	Assisted	Total
Sex			
Male	125 (51.9)	116 (48.1)	241 (84.0)
Female	18 (40.9)	26 (59.1)	44 (15.3)
Missing	2 (100)	0	2 (0.7)
Region			
Dar es Salaam	83 (50.9)	80 (49.1)	163 (56.8)
Dodoma	13 (27.1)	35 (72.9)	48 (16.7)
Mara	49 (64.5)	27 (35.5)	76 (26.5)
Age group (years)			
18 to <40	100 (51.0)	96 (49.0)	196 (68.3)
≥40	43 (48.3)	46 (51.7)	89 (30.0)
Missing	2 (100)	0	2 (0.7)
Highest educational level attained			
Cannot read and write	1 (7.7)	12 (92.3)	13 (4.5)
Can read only	73 (48.3)	78 (51.7)	151 (52.6)
Primary	54 (55.7)	43 (44.3)	97 (33.8)
Secondary	15 (62.5)	9 (37.5)	24 (8.4)
Missing	2 (100)	0	2 (0.7)

Values are reported as *n *(%).

### Cross-sectional surveys.

The cross-sectional baseline survey was conducted between June 2 and June 21, 2022, and the end-line survey was conducted between September 5 and September 27, 2022.

A total of 1,237 individuals were approached for participation in the baseline assessment, of whom 1,206 (97.5%) consented to participate in the survey. Participants’ baseline survey characteristics are presented in Supplemental Table 1. In both the control and intervention groups, more females (*P*-value <0.001) participated in the survey. More participants in the intervention group attained secondary education compared with the control group (40% versus 23.8%; *P*-value <0.001), and more than 65% of participants were engaged in business. Less than 6% were working in the mining sector, and as expected because of the location of the tents, there were fewer (2.7%) drivers in the control group compared with the intervention group (10.2%; *P*-value <0.001).

For the end-line survey, a total of 1,208 participants were contacted, of whom 1,167 (96.6%) provided consent. The end-line survey participant characteristics are provided in Supplemental Tables 1 and 2. Again, there were significantly (*P*-value = 0.006) more female participants than male participants in both the control and intervention groups. The majority (63.8%) of participants attained primary education (73.1% in control and 54% in intervention [*P*-value <0.001]). More participants (65.9% versus 56.5%; *P*-value <0.001) in the intervention group were doing business compared with the control group. In the control group, 6.6% were engaged in the mining sector, compared with 2.3% in the intervention group (*P*-value <0.001). As before and as expected, there were fewer (5%) drivers in the control group compared with the intervention group (11.2%; *P*-value <0.001; Supplemental Table 1). More participants (*P*-value <0.001) were in the age group of 18 to <40 years during the end-line survey. Approximately 10% more participants during the end-line survey attained primary education (*P*-value = 0.003).

Baseline participants’ responses show that more than 98% of participants were willing to test with a nasal Ag-RDT. The majority (>75%) of participants indicated that they would prefer to self-test. Most (64.4%) would test for self-protection, and only 7.6% would test to stop transmission in the community. A total of 1,055 (87.5%) of participants thought it was necessary to test for COVID-19 before being vaccinated. Overall, at baseline, the majority (∼63%) of participants indicated that they had no previous information on the availability of COVID-19 Ag-RDTs (Table 4).

**Table 4:** Willingness to test with Ag-RDTs

Variable	Baseline				End-Line			
	Control	Intervention	Total	*P*-Value	Control	Intervention	Total	*P*-Value
Reason it is necessary to test for COVID-19								
To protect others	231 (38.4)	189 (31.2)	420 (34.8)	0.009	154 (30.7)	147 (29.9)	301 (27.2)	0.784
To know COVID-19 status	295 (49.1)	348 (57.5)	643 (53.3)	0.003	310 (61.8)	177 (36)	487 (44)	<0.001
For travel purposes	18 (3)	6 (1)	24 (2)	0.013	14 (2.3)	15 (2.6)	29 (2.6)	0.746
To stop transmission in the community	49 (8.2)	43 (7.1)	92 (7.6)	0.429	28 (4.7)	37 (6.5)	65 (5.9)	0.175
For self-protection	391 (65.1)	386 (63.8)	777 (64.4)	0.649	294 (49.2)	435 (76.5)	729 (65.9)	<0.001
Other	22 (3.7)	13 (2.2)	35 (2.9)	0.118	19 (3.2)	10 (1.8)	29 (2.6)	0.119
Thought it is necessary to test for COVID-19 before being vaccinated	524 (87.2)	531 (87.8)	1,055 (87.5)	0.746	534 (89.3)	515 (90.5)	1,049 (94.8)	0.011
Thought community has no sufficient information on availability of Ag-RDT nasal swab self-test for COVID-19	348 (57.9)	416 (68.8)	764 (63.4)	<0.001	307 (51.3)	316 (55.5)	623 (53.4)	<0.001
Willing to have a COVID-19 rapid test and wait for the results for 20 minutes	599 (99.7)	601 (99.3)	1,200 (99.5)	0.687	578 (96.7)	551 (96.8)	1,129 (96.7)	0.862
Willing to do the test through								
Self-testing	461 (76.7)	497 (82.2)	958 (79.4)	0.019	358 (61.6)	238 (43.0)	596 (52.6)	–
Assisted testing	140 (23.3)	108 (17.9)	248 (20.6)		223 (38.4)	315 (57.0)	538 (47.4)	<0.001
Things that will mobilize the use of nasal Ag-RDT for COVID-19 self-testing								
Free provision	182 (30.3)	137 (22.6)	319 (26.5)	0.003	149 (24.9)	101 (17.8)	250 (22.6)	0.003
Cheap price	114 (19)	174 (28.8)	288 (23.9)	<0.001	120 (20.1)	85 (14.9)	205 (18.5)	0.021
Easy to use	68 (11.3)	110 (18.2)	178 (14.8)	0.001	107 (17.9)	63 (11.1)	170 (15.4)	0.001
Ready availability	63 (10.5)	86 (14.2)	149 (12.4)	0.049	86 (14.4)	135 (23.7)	221 (20)	<0.001
Provision of education to the community	372 (61.9)	463 (76.5)	835 (69.2)	<0.001	381 (63.7)	460 (80.8)	841 (76)	<0.001
Other	51 (8.5)	58 (9.6)	109 (9)	0.505	24 (4)	43 (7.6)	67 (6.1)	0.009
Willingness to test with nasal Ag-RDT to protect loved ones								
Willing	590 (98.2)	596 (98.5)	1,186 (98.3)	–	575 (96.2)	551 (96.8)	1,126 (96.5)	–
Not sure	7 (1.2)	5 (0.8)	12 (1.0)	0.884	11 (1.8)	10 (1.8)	21 (1.9)	0.727
Unwilling	4 (0.7)	4 (0.7)	8 (0.7)	–	12 (2)	8 (1.4)	20 (1.8)	–

Ag-RDT = antigen rapid diagnostic test; COVID-19 = coronavirus disease 2019. Values are reported as *n *(%). *P*-values were calculated by using χ^2^ tests.

End-line participants’ responses show that more than 96% of participants were willing to test with a nasal Ag-RDT. The majority (61.6%) of participants in the control group indicated that they would prefer to self-test, compared with 43% in the intervention group (*P*-value <0.001). More participants (76.5%) in the intervention group said they would test for self-protection, compared with 49.2% in the control group (*P*-value <0.001), and only 6% indicated they would test to stop transmission in the community. A total of 1,049 (94.8%) participants reported that it was necessary to test for COVID-19 before being vaccinated. The majority of participants (96.7%) were willing to have a COVID-19 rapid test and wait for 20 minutes for the results.

**Figure 3.**
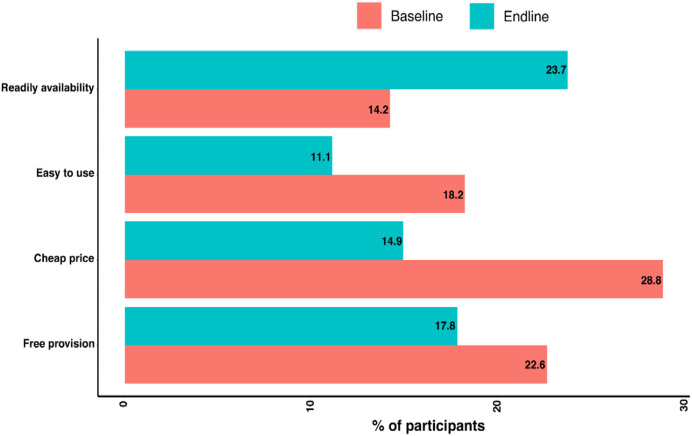
Participants’ opinions (in intervention group, at baseline and end-line survey) on the things that will mobilize the use of nasal antigen rapid diagnostic tests for self-testing of coronavirus disease 2019.

There was a significant difference (*P*-value <0.001) in the willingness to self-test between the baseline survey (82.2%) and end-line survey (43%) in the intervention group (Table 4). Figure 3 presents the intervention group participants’ opinions between the baseline and end-line surveys on the things that will mobilize the use of nasal Ag-RDT for the self-testing of COVID-19. There was a statistically significant difference (*P*-value <0.05) between the baseline and end-line surveys (in the intervention group) with regard to which factors would increase the use of nasal Ag-RDTs for COVID-19 self-testing, notably for the price and ready availability of the test (Figure 3).

**Figure 4.**
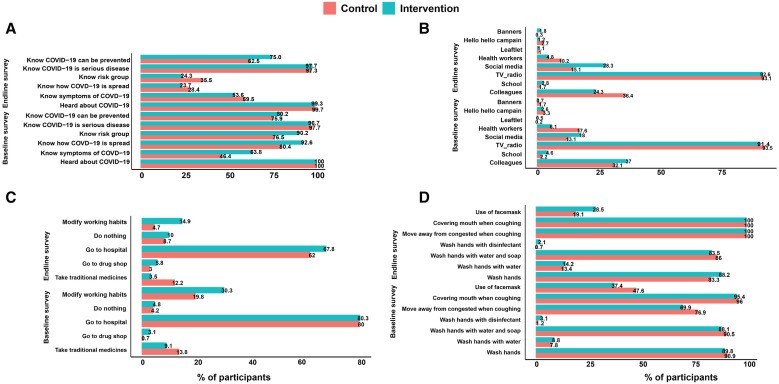
Knowledge, attitudes, and practice related to antigen rapid diagnostic tests.

We next assessed the knowledge, attitude, and practice related to Ag-RDT for the control and intervention as part of the baseline and end-line surveys (Figure 4). Overall, 941 (78%) participants thought COVID-19 could be prevented, and 97.2% understood COVID-19 to be a serious disease. A total of 967 (80.2%) said they would go to the hospital if they were in contact with a COVID-19-suspected patient, whereas 4.5% indicated that they would do nothing. All participants interviewed had heard about COVID-19 disease for both the baseline and end-line surveys. The baseline participants’ responses show that the TV or radio was the main source of information for both the control and intervention groups (93.5% versus 91.4%). Participants in the intervention group knew more about symptoms of the disease (63.8% versus 46.4%; *P*-value <0.001), how the disease spreads (92.6% versus 80.4%; *P*-value <0.001), and which groups are at a higher risk (90.2% versus 76.5%; *P*-value <0.001; Figure 4).

For the end-line participants, the TV or radio was also mentioned to be the main source of information. Overall, 92.4% heard about COVID-19 disease from the TV or radio. Approximately 12% (36.4% versus 24.3%) and 5% (10.2% versus 4.8%) more participants in the control group compared with intervention got information on COVID-19 from colleagues and health workers, respectively (*P*-value <0.001), whereas ∼13% (28.3% versus 15.1%) more participants in the intervention group, compared with the control, got information from social media (*P*-value <0.001). In contrast to the baseline survey, the majority of participants in the control group knew about symptoms of the disease, compared with the intervention group (59.5% versus 53.6%; *P*-value = 0.041), and knew more about which population group was at a higher risk (35.5% versus 24.3%; *P*-value <0.001). As before, participants of the intervention group were more aware of how the spread of COVID-19 can be prevented (75.0% versus 62.5%; *P*-value <0.001). The majority of participants (67.8% in the intervention group and 62% in the control group) indicated that they would go to the hospital if they had been in contact with a patient suspected to have COVID-19. Approximately 10% (14.9% versus 4.7%) more participants (*P*-value <0.001) in the intervention group compared with the control reported that they would change their working habits for fear of getting COVID-19 (Figure 4).

### COVID-19 vaccination status.

**Figure 5.**
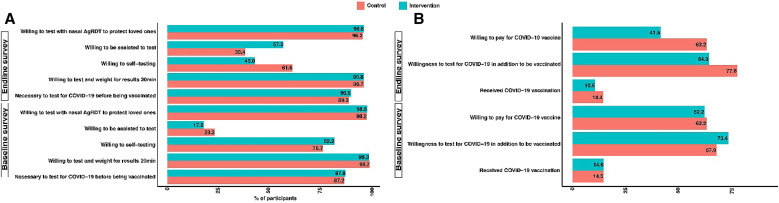
Willingness and perception toward coronavirus disease 2019 (COVID-19) antigen rapid diagnostic testing and COVID-19 vaccination.

Overall, during the baseline survey, 62.7% of the 1,206 participants surveyed were willing to pay for the vaccine. Responses from the baseline participants show that only ∼15% of participants received a COVID-19 vaccination, whereas responses from the end-line participants show that only 12.5% of participants received a COVID-19 vaccination. Figure 5 presents willingness and perception toward COVID-19 Ag-RDT testing and COVID-19 vaccination for control and intervention during baseline and end-line surveys. During the baseline survey, more participants in the intervention group (82.2%) were willing to self-test for COVID-19, compared with 76.7% in the control group (*P*-value <0.001). However, this changed during the end-line survey, when more participants in the control group (61.6%), compared with 43.0% in the intervention, were willing to self-test for COVID-19 (*P*-value <0.001). During the baseline survey, more participants in the intervention group (73.4%) were willing to test for COVID-19, in addition to vaccination, compared with 67.9% in the control group (*P*-value = 0.036). Whereas during the end-line survey, more participants in the control group (77.6%), compared with 64.3% in the intervention, were willing to test for COVID-19, in addition to vaccination (*P*-value <0.001). There was no difference (*P*-value = 0.698) in the willingness to pay for the vaccine between the control and intervention groups during the baseline survey; however, during the end-line survey, participants in the control group were more willing (63.2% versus 41.5%; *P*-value <0.001) to pay for the vaccine compared with the intervention group.

### Comparison between baseline and end-line surveys for the intervention areas.

Overall, considering only the intervention areas, there were small differences between the baseline and end-line surveys in the various parameters assessed. The majority of the participants knew about COVID-19 and the intervention measures, although COVID-19 Ag-RDT was not known to most of them, even during the end-line survey. Notably, at both the baseline and the end-line, most participants were willing to have a COVID-19 rapid test and wait for the results for 20 minutes (99.3% versus 96.8%, respectively). Similarly, most participants thought it was necessary to test for COVID-19 before vaccination in both the baseline and end-line surveys (87.8% versus 90.5%, respectively; *P*-value = 0.041; Figure 5). Other aspects, such as awareness of symptoms, appear to have been influenced by several confounding factors because several changes were also observed within control communities. Furthermore, the study was conducted at a time when SARS-CoV-2 transmission rates were low, and significant COVID-19 fatigue was observed. This influenced factors such as willingness to modify habits for fear of getting COVID-19 (30.3% at baseline versus 14.9% at the end-line survey in the control group and 19.8% at baseline versus 4.7% at the end-line survey in the intervention group), moving away from a congested place when coughing or sneezing (69.9% baseline versus 100% at the end-line for the control group and 76.9% at baseline versus 100% at the end-line survey in the intervention group), covering their mouth when coughing or sneezing (95.4% baseline versus 100% at the end-line for the control group and 96.0% at baseline versus 100% at the end-line survey in the intervention group), and using a face mask (37.4% baseline versus 28.5% at the end-line for the control group and 47.6% at baseline versus 19.1% at the end-line survey in the intervention group; Figure 4).

## DISCUSSION

The rollout of assisted COVID-19 Ag-RDT and self-testing was well-accepted in urban and rural communities, and reference was made to similar testing strategies for other indications, including pregnancy and HIV.^12^^–^^14^ The acceptability of community-based COVID-19 self-testing has been reported elsewhere.^10^^,^^15^^,^^16^ The uptake of Ag-RDT testing was high in all age groups. The fact that there were more younger participants is consistent with the existing population age structure within the participating communities.^17^ Younger participants were also more likely to test themselves than older participants. This finding is consistent with a recent study by Stohr et al.,^18^ who found that a higher age was independently associated with a false-negative self-testing result (odds ratio = 1.024 (95% CI 1.003–1.044)). More males participated in the public service delivery points than females. This may be attributed to the geographical location of the tents or the fact that fewer women are found in those locations compared with markets, for example. This suggests that any Ag-RDT implementation program should be spread across geographical locations representative of the targeted population. For instance, preference for house-to-house COVID-19 testing was also reported in another study conducted in Asia.^16^ Although for our study, concerns were raised during the stakeholder engagement meeting with the MoH on COVID-19 self-testing in terms of community adherence to the proper waste disposal guidelines after testing, how the individuals will deal with the positive results, and the decision to isolate or visit the nearby health facilities, these issues did not pose a challenge during the study. The IFU was translated into the local language, Swahili; the waste disposal instructions were included in the IFU; and the participants were sensitized to follow the COVID-19 recommended action of visiting nearby facilities for proper medical care.

Only ∼30% of study participants preferred to test themselves, and there is still a significant proportion of individuals who opted to be assisted during testing. As expected, those with lower education levels and those with advanced age were more likely to seek assistance.^10^ Preference for assisted testing may be due to low confidence in performing the test, uncertainty about what to do with a positive test result, and the unavailability of effective treatments for mild and moderate illness. In our logistic regression model, we found those who attended primary and secondary school had a 21% and 84% higher probability of testing themselves compared with those who did not attend school, respectively; however, this difference was not statistically significant.^18^ Stohr found similar results. Policymakers and health workers should create messaging for the community that could improve understanding of the test results and actions to be taken afterward.

Despite the acceptability of Ag-RDT use, the testing volume was much lower than anticipated because of the restrictions placed by the MoH eligibility criteria and rapidly declining COVID-19 transmission and illness during the period of study implementation. The prevalence of COVID-19 detected using the Ag-RDT was less than 1% in our study population, which is consistent with the low prevalence in the general community at that time.^19^ The reported consistently low coverage of COVID-19 vaccination in the study period also indicates that demand for all interventions was low, and a higher uptake of both diagnosis and vaccination can be expected in periods or areas of higher transmission.

This highlights that any strategy must take into account the change in COVID-19 transmission dynamics, especially if it relates to testing in the community.^20^ The MoH should also adopt a cautious and data-driven approach and response strategies that can help mitigate the risk of a resurgence, not only to COVID-19 but also to other respiratory diseases that may pose a threat to communities.

Generally, there was no major difference in various parameters assessed between the baseline and end-line surveys, and this is most likely attributed to the short implementation period and the decline in SARS-CoV-2 transmission rates. Importantly, there was no improvement seen in the preference of the community to self-test during the period. This may be due to the limited and short-lived community engagement activities and limited communication campaigns. At the end-line survey, most respondents still reported that they were not knowledgeable about Ag-RDTs. Most respondents reported to have received information on COVID-19 disease and intervention through mass media (TV and radio). The pattern of sources of information did not change between the baseline and the end-line survey period, indicating that TV and radio are the best methods of communicating information to the community. In this study, the importance of mass media in the dissemination of COVID-19-related messages could not be overemphasized, and its contributions have been reported by others.^21^^–^^23^

This study also aimed to improve vaccination coverage through testing campaigns. Although there were no significant changes in the reported vaccination coverage, it could be inferred from the results that the willingness to be vaccinated was high. A high number of individuals thought it was important to be tested before being vaccinated. Therefore, there are possibly many other factors that still discourage individuals from getting vaccinated.^24^^,^^25^ The low uptake of the vaccine in these communities, among other reasons, was due to mistrust of the COVID-19 vaccine because of unawareness of side effects, safety, and religious beliefs, as indicated in our previously published study.^26^ Moreover, the short study implementation period and small study areas with no mass communication strategies used could have impacted the low coverage of information communication and health education on the benefits of the COVID-19 vaccine to the community. Intensifying community sensitization and stakeholder engagement at different levels and age groups, paying attention to the risk and vulnerable populations, on the advantage of the vaccine has been shown to increase vaccination coverage.^27^^–^^29^ Therefore, the involvement of communities and stakeholders earlier on and frequent meetings should be considered in future studies to increase vaccine coverage.

The acceptability and uptake of Ag-RDT self-tests in the community increase confidence in the MoH to increase the accessibility of the test to the lower-level HCFs for prompt response against epidemics. In addition to vaccination, the availability of Ag-RDT diagnostic tests for COVID-19 for prompt detection and confirmation will strengthen Tanzania’s capacity to respond to and quickly mend the negative impact of the outbreaks. Moreover, community empowerment and their participation in disease control complement the frontline health workers, who are overstretched, especially in African countries.

The information system established for collecting data from the community worked well, and a good proportion of clients reported the results using that system, with slightly more than half of the test results reported. Some optimization has already been performed to improve the capture of critical information, and there are prospects that a higher proportion of reporting can be expected in the future. The systems seem very attractive for capturing the surveillance data of other emerging pathogens beyond COVID-19, and there were already requests to do so, for example, for influenza-like illnesses. Currently, there are some limitations to the application because it does not provide information on where the nearby service points are available or existing treatment options.

This study was conducted in both rural and urban areas with different social, economic, and cultural contexts. The approach we used to introduce self-testing can therefore be replicated in a variety of settings. Our findings are comparable with the study conducted by Mukoka et al.^10^

The limitations of our study include the short period for the pre- and post-survey and the small sample size, which is not enough to generalize the findings to other communities in Tanzania and African countries with diverse sociocultural contexts and demographic groups. Although self-testing was well-accepted in these three regions, either through self-test or assistance, to ensure higher acceptance by the general community and other African countries, further larger studies with longer periods of observation to evaluate the impact of the interventions and diverse populations, including cost-effectiveness and targeted populations, for the use of self-tests, especially during epidemics or pandemics, will fill the remaining knowledge gaps and inform the development of self-test guidelines. Another limitation was the risk of information bias, in which respondents could provide what they believe to be socially acceptable answers rather than the truth, especially with regard to questions asking about their behavior and health conditions. Finally, the data capture tools that were used (USSD) were not familiar to some participants, which imposed some challenges in using and sending the data. Further evaluation of the USSD usability and efficiency in data collection from the community before its interaction with the event-based surveillance system is warranted.

## CONCLUSION

COVID-19 self-testing and assisted testing were well-accepted in areas in which the Ag-RDT devices were deployed. With these findings, we recommend the MoH to integrate self-testing into existing health systems or campaigns in Tanzania to increase healthcare access and promote proactive health management within communities. However, successful integration requires careful planning, including considerations for accessibility, affordability, and education around proper testing procedures and result interpretation. Moreover, the COVID-19 testing manuals developed during project implementation and now approved to be used by the MoH will support the process because they provide information such as training, usage, waste management, and result interpretation. During the process, Tanzania regulatory authority guidelines for importation and distribution must be followed. Moreover, strategies and lessons learned from the introduction of HIV self-tests in the country can be adapted to support the implementation of COVID-19 self-testing in the communities. Self-testing should be supplemented with assisted testing, preferably using the community HCWs. The involvement of the community HCWs in testing and reporting cases will dramatically improve the completeness of community data reporting and enable an early warning system for preparedness and response against emerging and re-emerging pandemics, even beyond COVID-19. The COVID-19 vaccination did not impact the uptake of testing, and the benefit of the Ag-RDT testing program on the uptake of vaccination was also not established. A demonstration of the impact of these types of interventions will require extensive education and communication campaigns over a longer period of time using the TV and radio, which have been shown to be the best way of getting the messages to the community.

## Supplemental Materials

10.4269/ajtmh.23-0732 Supplemental Materials
