# Supplementary material for: Acceptability, Feasibility, and Uptake of COVID-19 Antigen Rapid Diagnostic Self-Testing at the Community Level in Tanzania
Source: Am J Trop Med Hyg. 2024 Nov 26;112(4 Suppl):26–36. doi: 10.4269/ajtmh.23-0732 (PMC11965726; doi:10.4269/ajtmh.23-0732)
Supplement: Supplemental Materials [file tpmd230732.SD1.pdf]

Table S1
Demographic information

Variable	Baseline				End-Line			
	**Control**	**Intervention**	**Total**	*P*-Value	**Control**	**Intervention**	**Total**	*P*-Value
Number of participants surveyed	601	605	1206		598	569	1167	
Sex								
Male	230 (38.3)	295 (48.8)	525 (43.5)	< 0.001	213 (35.6)	247 (43.4)	460 (39.4)	0.006
Female	371 (61.7)	310 (51.2)	681 (56.5)		385 (64.4)	322 (56.6)	707 (60.6)	
Region								
Dar-es-Salaam	199 (33.1)	206 (34.0)	405 (33.6)		200 (33.4)	200 (35.2)	400 (34.3)	
Dodoma	202 (33.6)	202 (33.4)	404 (33.5)	0.937	201 (33.6)	190 (33.4)	391 (33.5)	0.798
Mara	200 (33.3)	197 (32.6)	397 (32.9)		197 (32.9)	179 (31.5)	376 (32.2)	
Age (year) - mean +-SD	41.5 +-15.2	40.7 +-15.8	41.1 +-15.5		37.3 (15.5)	35.8 (14.3)		
18–<40	300 (49.9)	324 (53.6)	624 (51.7)	0.206	378 (63.2)	375 (65.9)	753 (64.5)	0.336
≥40	301 (50.1)	281 (46.4)	582 (48.3)		220 (36.8)	194 (34.1)	414 (35.5)	
Marital status								
Single	114 (19)	172 (28.4)	286 (23.7)		137 (22.9)	180 (31.6)	317 (27.2)	
Married/Co-habited	418 (69.6)	369 (61)	787 (65.3)	0.001	408 (68.2)	337 (59.2)	745 (63.8)	0.003
Widowed	62 (10.3)	53 (8.8)	115 (9.5)		40 (6.7)	33 (5.8)	73 (6.3)	

Divorced	7 (1.2)	11 (1.8)	18 (1.5)		13 (2.2)	19 (3.3)	32 (2.7)	
Highest educational level attained								
None	58 (9.7)	48 (7.9)	106 (8.8)		33 (5.5)	31 (5.5)	64 (5.5)	
Primary	358 (59.6)	267 (44.1)	625 (51.8)	< 0.001	437 (73.1)	307 (54)	744 (63.8)	< 0.001
Secondary	143 (23.8)	242 (40.0)	385 (31.9)		105 (17.6)	181 (31.8)	286 (24.5)	
College and above	42 (7)	48 (7.9)	90 (7.5)		23 (3.9)	50 (8.8)	73 (6.3)	
Employment status								
Employed	8 (1.3)	16 (2.6)	24 (2)		3 (0.5)	14 (2.5)	17 (1.5)	
Unemployed	29 (4.8)	41 (6.8)	70 (5.8)	0.05	11 (1.8)	15 (2.6)	26 (2.2)	
Self employed	86 (14.3)	110 (18.2)	196 (16.3)		215 (36)	126 (22.1)	341 (29.2)	< 0.001
Business	432 (71.9)	393 (65)	825 (68.4)		338 (56.5)	375 (65.9)	713 (61.1)	
Other	46 (7.7)	45 (7.4)	91 (7.6)		31 (5.2)	39 (6.9)	70 (6)	
Occupation/Trade								
Mining	31 (5.7)	31 (5.6)	62 (5.6)		25 (6.6)	10 (2.3)	35 (3)	
Farmer	130 (23.7)	79 (14.2)	209 (18.9)		111 (29.2)	105 (24.5)	216 (18.5)	
Food vendors	79 (14.4)	91 (16.3)	170 (15.4)	< 0.001	58 (15.3)	85 (19.8)	143 (12.3)	< 0.001
Drivers	15 (2.7)	57 (10.2)	72 (6.5)		19 (5.0)	48 (11.2)	67 (5.7)	
Other	293 (53.5)	299 (53.7)	592 (53.6)		167 (44)	181 (42.2)	348 (29.8)	

Values are reported as *n* (%), *P*-value was calculated from Chi-square test.

Table S2

Demographic information for the intervention areas

**Variable**	**Baseline**	**End-line**	**Total**	*P*-Value
Number of participants surveyed	605	569	1174	
Sex				
Male	295 (48.8)	247 (43.4)	542 (46.2)	0.066
Female	310 (51.2)	322 (56.6)	632 (53.8)	
Region				
Dar-es-Salaam	206 (34.0)	200 (35.2)	406 (34.6)	0.899
Dodoma	202 (33.4)	190 (33.4)	392 (33.4)	
Mara	197 (32.6)	179 (31.5)	376 (32)	
Age (year) - mean +-SD	40.7 +-15.8	35.8 (14.3)		
18 - < 40	324 (53.6)	375 (65.9)	699 (59.5)	<0.001
>= 40	281 (46.4)	194 (34.1)	475 (40.5)	
Marital status				
Single	172 (28.4)	180 (31.6)	352 (30)	0.062
Married/Co-habited	369 (61)	337 (59.2)	706 (60.1)	
Widowed	53 (8.8)	33 (5.8)	86 (7.3)	
Divorced	11 (1.8)	19 (3.3)	30 (2.6)	
Highest educational level attained				

None	48 (7.9)	31 (5.5)	79 (6.7)	
Primary	267 (44.1)	307 (54)	574 (48.9)	0.003
Secondary	242 (40.0)	181 (31.8)	423 (36)	
College and above	48 (7.9)	50 (8.8)	98 (8.3)	
Employment status				
Employed	16 (2.6)	14 (2.5)	30 (2.6)	
Unemployed	41 (6.8)	15 (2.6)	56 (4.8)	
Self employed	110 (18.2)	126 (22.1)	236 (20.1)	0.011
Business	393 (65)	375 (65.9)	768 (65.4)	
Other	45 (7.4)	39 (6.9)	84 (7.2)	
Occupation/Trade				
Mining	31 (5.6)	10 (2.3)	41 (4.2)	
Farmer	79 (14.2)	105 (24.5)	184 (18.7)	
Food vendors	91 (16.3)	85 (19.8)	176 (17.8)	<0.001
Drivers	57 (10.2)	48 (11.2)	105 (10.6)	
Other	299 (53.7)	181 (42.2)	480 (48.7)	

Values are reported as *n* (%), *P*-value was calculated from Chi-square test.
